# Evaluation of the applicability of internal controls on self-collected samples for high-risk human papillomavirus is needed

**DOI:** 10.1186/s12905-023-02691-8

**Published:** 2023-11-27

**Authors:** Bo Verberckmoes, Tamara De Vos, Karel Maelegheer, Catherine Ali-Risasi, Yolande Sturtewagen, Marleen Praet, Davy Vanden Broeck, Elizaveta Padalko

**Affiliations:** 1 Department of Public Health and Primary Care, Ghent University, International Centre for Reproductive Health (ICRH), Corneel Heymanslaan 10, Ghent, 9000 Belgium; 2 Department of Diagnostic Sciences, Ghent University, Corneel Heymanslaan 10, Ghent, 9000 Belgium; 3 Algemeen Ziekenhuis Sint-Lucas Brugge, Sint-Lucaslaan 29, Brugge, 8310 Belgium; 4 Department of Clinical Biology, General Provincial Hospital of Kinshasa, Avenue Colonel Ebeya 169, Kinshasa, Democratic Republic of Congo; 5 Laboratory of Molecular Pathology, Algemeen Medisch Labo, Antwerp, Belgium; 6 Department of Obstetrics and Gynaecology, Ghent University Hospital, Corneel Heymanslaan 10, Ghent, 9000 Belgium

**Keywords:** Human papillomavirus, Self-sampling, Internal control, HPV assay

## Abstract

**Background:**

Self-collection of cervical samples to detect high-risk human papillomavirus (hr-HPV) is a trending topic in primary cervical cancer screening. This study evaluates the applicability of a self-sampling device to routine molecular procedures for hr-HPV detection.

**Methods:**

In a primary health care facility in Kinshasa, Congo, 187 self-collected samples (Evalyn Brush) were gathered and sent to Ghent University Hospital (UZ Ghent) and Algemeen Medisch Labo (AML) in Belgium where routine tests for hr-HPV were applied (Abbott RealTime hr-HPV and qPCR (E6/E7), respectively). Sample type effect was evaluated by comparing the internal control (IC) between the self-collected samples and routine, clinician-taken samples randomly selected from the UZ Ghent archive.

**Results:**

In UZ Ghent an error was encountered in 9.1% (17/187) of self-collected samples due to a lack of IC signal. The hr-HPV prevalence in the remaining 170 samples was 18,8%. Comparing IC results between the self-collected and clinician-collected groups, a significant difference (p < 0,001) was found, with higher IC signals in the clinician-collected group. In AML, an error was encountered in 17.6% (33/187) of samples, including 16/17 of the UZ Ghent. The remaining sample with IC error gave a negative result in AML. Among the 154 samples without IC error at AML, a correlation of 90% was seen between both laboratories with a 77% negativity rate.

**Conclusion:**

Testing the self-collected specimens by 2 routine hr-HPV tests gave a high IC error rate (9.1–17.6%). A possible solution would be to differentiate cut-offs for IC values depending on sample type, as currently used cut-offs are set for clinician-taken samples.

**Supplementary Information:**

The online version contains supplementary material available at 10.1186/s12905-023-02691-8.

## Introduction

Cervical cancer screening programs have already reduced the incidence and mortality rate of cervical cancer in industrialized countries [1]. Many of these screening programs use cervical cytology screening as the primary test. Recent data show that high-risk human papillomavirus (hr-HPV) testing is more effective as a primary test in reducing cervical cancer incidence and mortality [2–5]. Furthermore, implementing hr-HPV testing as the primary screening tool opens up the possibility of performing screening on self-collected samples. The use of self-collected (cervico-)vaginal samples as an alternative for the clinician-collected samples for screening could improve participation among the nonresponders in countries with a well-established screening program [6]. It is known that nonattendance is the main reason for the remaining cases of highly invasive cervical carcinoma [7]. Offering self-sampling as an additional option in a screening program increases the participation rate [8]. Most women even prefer self-sampling over traditional screening, because of advantages such as time, place of sampling and privacy issues [9].

For developing countries, where organized screening programs are lacking due to their high cost and the limited health infrastructure, self-collection and primary HPV testing offer a solution for the low screening rates [10].

Currently, we have an abundance of HPV tests on the global market. The vast majority of them lack proper evaluation in line with consensus requirements, nor are they validated to be used on various clinical specimen types [11]. Each different combination of device, buffer and assay/system requires validation, either by the manufacturer or by individual laboratories. While most assays present data from the scientific literature indicating that their assays perform well with self-collected specimens, there does not appear to be any clinically validated assay with widely accepted (e.g., CE mark or FDA-approved) instructions for use covering self-collected specimens [12].

Despite a growing number of studies on self-collection and HPV testing, little attention has been directed towards the compatibility and alignment of the existing assays with these self-sampling devices. In terms of sensitivity, self-collection is comparable to current screening practices for detecting cervical carcinoma and high-grade lesions, but device and specimen processing effects exist [13]. Studies have also shown that the type of hr-HPV assay used is more important than the type of sampling device [14, 15]. In light of this matter, Arbyn et al. designed a protocol, the VALHUDES protocol, which offers a framework for validation of HPV assay and self-sample device combinations [16].

Another critical point when shifting to self-collection is the sample adequacy. Unfortunately, not all HPV assays incorporate a sample adequacy control (SAC) or internal control (IC) [17]. Without SAC, the cellularity of the sample cannot be monitored, the confidence in a negative result remains questionable and the efficacy of screening is compromised [18].

The aim of the present study was to evaluate the applicability of the Evalyn self-sampling device to two routine molecular procedures for hr-HPV detection.

## Materials and methods

### Study population

In total, 187 self-samples were collected among women in a primary health care facility in Kinshasa, Congo. All women were between 29 and 73 years old (mean 38,7 y).

### Self-sampling device

All women received an Evalyn brush (Rovers Medical Devices B. V., Oss, the Netherlands) to perform self-sampling. When receiving this device, they were given oral information and a flyer about how to use it. The Evalyn brush is considered user-friendly [19]. This device is approximately 20 cm long and has lateral wings controlling the depth of insertion. After insertion, the plunger has to be pushed to extend the brush and then rotated for five times to collect the sample. Every rotation is indicated by a clicking sound. After self-sampling, the plunger is pulled back into the casing and a cap is placed over the brush, after which the sample can be sent by mail at room temperature. This device is a dry storage system. When it was delivered to the laboratory, the Evalyn brush was suspended in 20 ml of ThinPrep (PreservCyt, Hologic, USA).

### Molecular procedures: HPV detection and internal control

For hr-HPV evaluation in UZ Ghent, the Abbott RealTime High Risk HPV test (RealTime; Abbott GmbH & Co. KG, Wiesbaden, Germany) was used to examine the samples. It is an automated, qualitative multiplex assay based on real-time polymerase chain reaction (PCR) for the detection of 14 hr-HPV genotypes (16, 18, 31, 33, 35, 39, 45, 51, 52, 56, 58, 59, 66 and 68) and genotyping for HPV 16 and 18. In the obtained results, the cycle numbers (CN) indicate the strength of the signal. The higher the CN, the weaker the signal, probably indicating a lower viral load. The Abbott test detects the endogenous human beta globin sequence as an internal control (IC) signal to evaluate cell adequacy, sample extraction and amplification efficiency. The CN cut-off for IC was set at 35 cycles, while for HPV detection, the CN cut-off was set at 32 cycles. When the CN is higher, the target (IC or HPV) is reported as undetected. In addition to the hr-HPV results, we collected the CN for every target in every sample.

In the second laboratory, Algemeen Medisch Labo (AML) in Antwerp, qPCR (E6/E7) was used for hr-HPV evaluation. It is a qualitative assay based on real-time PCR for the detection and genotyping of 18 HPV genotypes, low- and high-risk (6, 11, 16, 18, 31, 33, 35, 39, 45, 51, 52, 53, 56, 58, 59, 66, 67, 68). It also detects the endogenous human beta globin sequence as an internal control (IC) signal to evaluate cell adequacy. The CN cut-off for the IC is set at 35 cycles; when it is between 30 and 35, it is reported as “low DNA”.

Considering we will compare the results of UZ Ghent with the results of AML, we exclude AML’s results of HPV 6/11/53/67 as these HPV genotypes are only detected by the AML-assay.

### Clinician-sampling control

All 239 clinician-taken samples were randomly selected from our routine archive at UZ Ghent. The samples were pap smears taken in routine care and stored in ThinPrep medium at room temperature.

### Statistical analysis

IBM SPSS Statistics version 28 was used for statistical evaluation and visualization. To evaluate whether data were normally distributed, the Shapiro-Wilk test was used in combination with a visual inspection using a histogram, boxplot and QQ-plot. If data were not normally distributed, it was retested for normality after log-transformation. Per HPV assay, HPV DNA-positive cases and cases of coinfection were determined. To evaluate the correlation between the two HPV assays, concordance in terms of HPV detection and IC signal among the two different HPV assays was assessed by a cross table comparison and by a Spearman correlation test (after exclusion of the samples with a nonreactive signal for the IC). The two assays were negatively agreeing if they both demonstrated a negative result for all hr-HPV genotypes. To evaluate the sample type effect, the CN of IC (UZ Ghent) of self-collected samples and the CN of IC of clinician-collected samples were compared by using the Mann-Whitney U test. P-values of less than 0.05 were considered statistically significant.

## Results

### Self-collected samples (UZ Ghent)

Of the 187 study samples, 17 samples (9,1%) showed an error in the first test run. Of these, 12 reported the error “the internal control was nonreactive” and five samples reported the error “the cycle numbers of the internal control were out of range”. In these last samples, the CN for the IC was higher than 35 but still detectable (range 35,10–36,45). In none of these 17 samples was a signal for hr-HPV types detected (Table 1).

In the samples without error, the hr-HPV prevalence was 18,8% (32/170). In the hr-HPV-positive group, 2/32 (6,3%) had an HPV 16 infection, 25/32 (78,1%) had an infection with at least one hr-HPV type other than HPV 16 and HPV 18, 3/32 (9,4%) had an infection with HPV 16 and at least one other hr-HPV and 2/32 (6,3%) had an infection with HPV 18 and at least one other hr-HPV (Table 1).

Of the 138 hr-HPV-negative samples, 16 showed a detectable signal for hr-HPV, with CN above the 32 cut-off. Of these, two samples reported a low signal for HPV 16, one sample for HPV 18 and 13 for another hr-HPV (Supplementary Table 1).

Four of the negative samples with an amplification curve suggestive of inhibition were diluted (1:3). However, two of these samples had a signal in the first test run but lost this signal after dilution probably because the viral load was diluted to such a level that the amplification signal was no longer detectable. The other two samples showed the same hr-HPV detection as before dilution, but the CN was higher. No interaction of inhibiting factors could be proven.

### Interlaboratory correlation

#### qPCR E6/E7 results for the self-collected samples (AML)

**Fig. 1:**
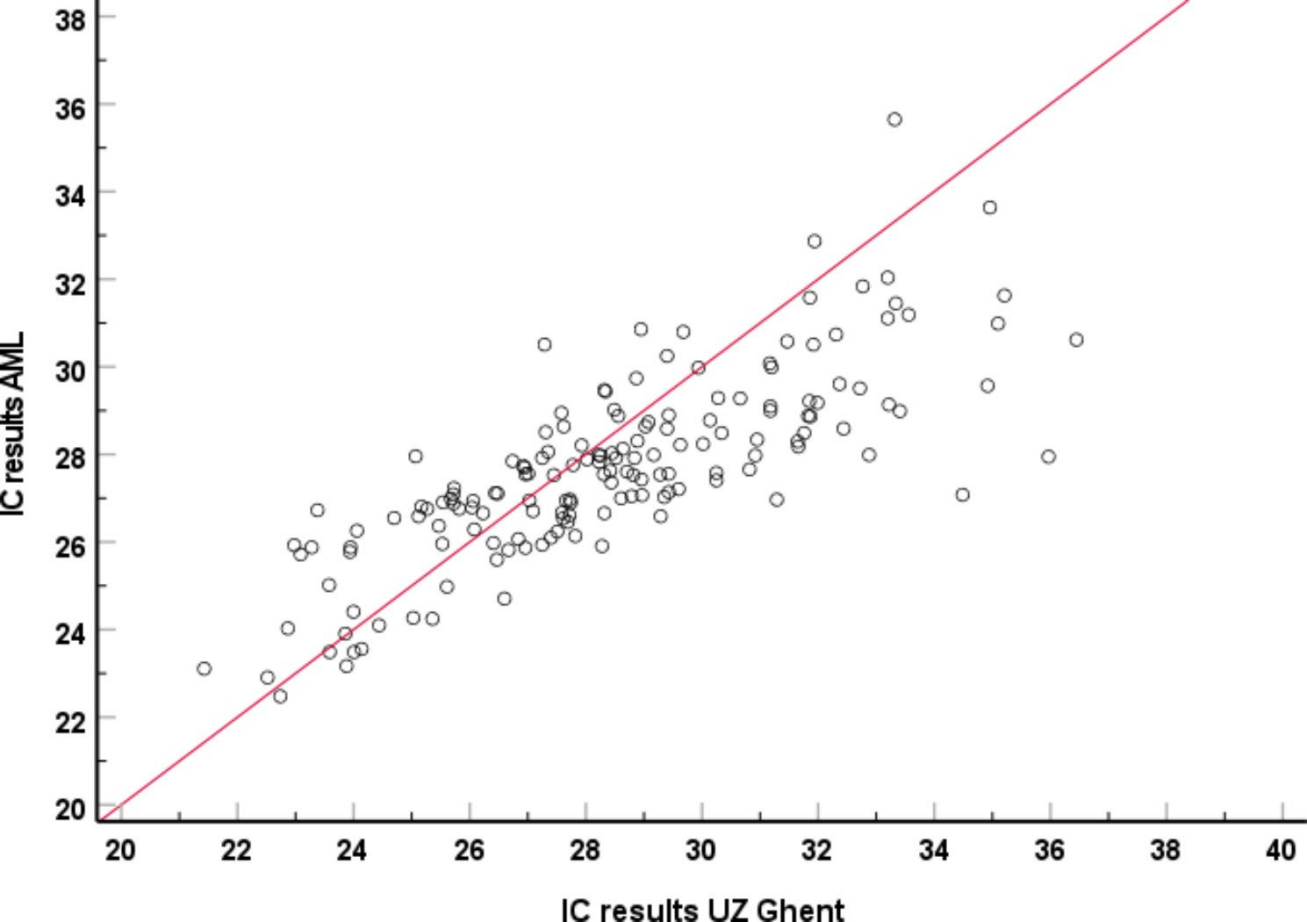
Correlation graph between IC results of UZ Ghent vs. AML

The IC values generated with the qPCR E6/E7 were not normally distributed (p < 0.001 and visual deviations from a normal distribution). In AML, an error was encountered in 17.6% (33/187) of samples, including 16/17 of the UZ Ghent IC errors. The remaining UZ Ghent IC error gave a negative result in AML (Table 1). Visually, there is a clear correlation between the IC results of UZ Ghent and AML (Fig. 1). In the remaining 154 samples a correlation of 90% (138/154) was seen between both laboratories with a 77% (118/154) negativity rate.

Of the 16 hr-HPV-negative samples with a signal for hr-HPV higher than the cut-off in UZ Ghent, only one had a hrHPV-positive result in AML (Table 1). This sample was positive for HPV 52 in AML and gave a signal (higher than the cut-off) for at least one or more other hr-HPV types in UZ Ghent.

**Table 1:** Comparison of hr-HPV prevalence and IC in self-collected samples assayed by Abbott RealTime hr-HPV PCR (UZ Ghent) and qPCR (E6/E7) (AML)

AML									Total
		Not detected	HPV16	Other hr-HPV	HPV16 & other	HPV18 & other	IC non- reactive	IC > 35CN	
**UZ Ghent**	**Not detected**	112	0	10	0	0	9	7	138
	**HPV16**	0	2	0	0	0	0	0	2
	**Other hr-HPV**	4	0	20	0	0	1	0	25
	**HPV16 & other**	0	0	0	3	0	0	0	3
	**HPV18 & other**	1	0	0	0	1	0	0	2
	**IC nonreactive**	0	0	0	0	0	12	0	12
	**IC > 35CN**	1	0	0	0	0	2	2	5
**Total**		118	2	30	3	1	24	9	187

Hr-HPV: high risk Human Papilloma virus, IC: internal control, CN: cycle numbers

### Sample type effect

#### IC comparison self-collected samples vs. clinician-collected samples

**Fig. 2:**
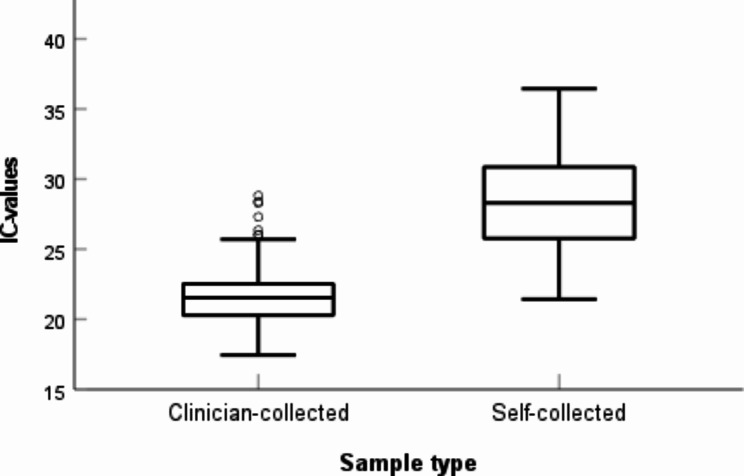
Comparison of CN values of IC signals between the self-collected and clinician-collected samples

Both IC-values of the self-collected and the clinician-collected group were not normally distributed (p < 0.001 and visual deviations from a normal distribution). Comparing IC between the self-collected and clinician-collected groups, a significant difference was seen (p < 0,001), with higher signals for IC (lower CN) in the clinician-collected group. The clinician-collected group had a mean of 21,61 CN, and the self-collected group had a mean of 26,69 CN (Fig. 2).

## Discussion

Implementing an option for self-sampling in the existing screening programs for cervical cancer is a way to improve screening coverage. As stated earlier, detecting hr-HPV types instead of using cytology screening as a primary screening tool could be more efficient. Questions arise whether assays with cut-offs often validated and optimized for specific circumstances can simply be applied to self-collected samples.

Many different types of self-sampling devices have been developed. All with differences in ease of use and performance. Enerly et al. compared the Delphi Screener, a lavage-based sampler, and the Evalyn brush. Samples were tested for HPV with the CLART HPV2 test (Genomica, Madrid, Spain) and the digene Hybrid Capture 2 (HC2) test (QIAGEN, Hilden, Germany). All samples contained enough biological material for testing. The overall agreement between the results of both tests was substantial but better for the Evalyn brush [8]. Jentschke et al. compared the Qvintip (Aprovix AB, Uppsala, Sweden), a dry brush, and Evalyn Brush. The samples were tested with the Abbott RT hr-HPV test. Four samples (two Evalyn brush samples, two Qvintip samples) did not contain enough cellular material (β-globin above threshold) [19]. Chen et al. performed a study comparing the clinical performance of self-sampling (Evalyn brush) with clinician sampling. Hr-HPV status was tested with real-time PCR technology. This study found excellent agreement between both sample types [20]. Neither the study of Jentschke et al. nor of Chen et al. took the amplification beyond the cycle number threshold into account [19, 20]. Leinonen et al. compared the Evalyn brush with the FLOQSwab for self-sampling (COPAN, Brescia, Italy). The samples were tested with Anyplex II HPV28 (Seegene Inc., Seoul, Korea), Cobas 4800 (Roche Molecular Diagnostics, Pleasanton, USA) and Xpert HPV (Cepheid, Sunnyvale, USA). Two hundred and thirty-two patients had a complete triplet of HPV test results for comparison. FLOQSwabs had more invalid results than Evalyn brushes. The clinical performance of both devices was noninferior to that of the physician-collected samples when processed within 4 weeks. Unfortunately, discordant pairs were not examined further in detail [13].

Snijders et al. concluded that the use of Qvintip resulted in a lower signal for cellular material than the use of Evalyn brushes. It did not, however, influence the clinical performance [21].

Differences in test results can also be caused by differences in the method of hr-HPV detection. Jentschke et al. found better clinical performance for hr-HPV detection with Abbott RealTime PCR than with HC2 on self-collected lavage samples [22]. In the meta-analysis by Arbyn et al., different HPV tests were compared, reporting the best clinical performance on self-collected samples with Abbott RealTime PCR and the GP5+/6 + test [23]. The update of that meta-analysis reports an equivalent accuracy of self-samples and clinician samples when used with hr-HPV assays based on PCR [15].

In this study, 187 self-collected samples of Congolese women, using the Evalyn brush, were tested for 14 different types of hr-HPV with Abbott in UZ Ghent and qPCR (E6/E7) in AML. In UZ Ghent, an error was reported for 17 of these samples, indicating that there was no or a low signal for cellular material. Sixteen of these showed the same result in Antwerp, and the other one showed a negative hr-HPV result. The samples with a low signal for cellular material may contain cells, but fewer than when clinicians take the sample. The samples without a signal for the internal control probably did not contain any cells or hardly any cells. It is easy to believe that in these cases, the women were not able to or did not dare to take the self-sampling in a correct way, meaning they did not collect any cervico-vaginal cells on the brush, resulting in a false-negative result. When, for instance, women had difficulties understanding the instructions and were not aware they had to push the plunger before rotating it, they would take the self-sample without extending the brush out of the casing. The results of these samples regarding HPV presence are not reliable because HPV positivity could be underestimated. It is possible that the number of errors could be reduced by offering a better explanation to the women about how to use the self-sampling device and making them feel confident in their ability to take the sample themselves. When considering implementing a self-sampling option in cervical cancer screening programs, this issue needs particular attention.

More studies are needed on what to do when the result is an error in IC and how to interpret IC values set for clinician-obtained cervical smears when applying them to self-samples. It can be suggested that women who provided a self-sample with a too low IC value will be requested to see a medical doctor to be taken a clinician-collected sample or given a second chance to self-sample. Unfortunately, the majority of HPV assays even lack an internal control or use controls that cannot challenge the cellularity of the specimen [17, 24]. This is a major limitation that must be taken into account when implementing a self-sampling-based cervical cancer screening program.

Overall, when using the same cut-off levels as for the clinical setting for which Abbott is produced, the hr-HPV-positive prevalence is 18,8%. Sixteen of the 17 samples with an error as a result gave an error in the results of AML. The remaining Ghent error gave a negative result in AML. Another 17 samples resulted in an error in the AML analysis. In the remaining 154 samples, a correlation of 90% was seen between both laboratories with a 77% negativity rate.

Considering that Abbott is produced for a clinical setting and clinician-collected cervical samples, we wondered if these cut-offs might need adjustment for the testing of self-collected samples of women in a screening setting. In a study by Bell et al., some cases were missed by the Abbott test using the manufacturer’s cut-offs. After adjustment of the cut-off for positivity to a less stringent one, the cases reported as negative nevertheless demonstrated the presence of hr-HPV but with associated Cq values above the manufacturer’s cut-off [25]. In our results, in the hr-HPV-negative group, 16/138 (11,6%) samples showed a detectable signal for hr-HPV, with CN above the 32 cut-off value. However, 15 of these 16 samples had a negative hr-HPV result in Antwerp, making it unlikely that these CNs above the 32 cut-off are in fact false-negatives. This needs further in-depth research.

## Conclusion

Implementing self-sampling in existing screening programs can potentially improve screening coverage. With regard to the current data, we suggest that further research should be focused on the evaluation of cut-offs for internal controls as well as HPV high-risk genotypes, specifically on self-collected samples. Therefore, there is surely an increasing need to adjust or confirm the cut-offs of internal controls to the application on self-collected samples that can be different regarding the self-sampling device and subsequent molecular test used.

### Electronic supplementary material

Below is the link to the electronic supplementary material.

Supplementary Material 1

## Data Availability

The datasets used and/or analysed during the current study are available from the corresponding author on reasonable request.

## List of abbreviations

Hr-HPV: high-risk human papilloma virus
UZ: Universitair Ziekenhuis (University Hospital)
AML: Algemeen Medisch Labo
qPCR: quantitative polymerase chain reaction
IC: internal control
SAC: sample adequacy control
CN: cycle numbers
Cq: quantification cycle

## References

[1] Lăără EDN, Hakama M (1987). Trends in mortality from cervical cancer in the nordic countries: association with organised screening programmes. Lancet.

[2] Ronco G, Giorgi-Rossi P, Carozzi F, Confortini M, Dalla Palma P, Del Mistro A (2010). Efficacy of human papillomavirus testing for the detection of invasive cervical cancers and cervical intraepithelial neoplasia: a randomised controlled trial. Lancet Oncol.

[3] Rijkaart DC, Berkhof J, Rozendaal L, van Kemenade FJ, Bulkmans NW, Heideman DA (2012). Human papillomavirus testing for the detection of high-grade cervical intraepithelial neoplasia and cancer: final results of the POBASCAM randomised controlled trial. Lancet Oncol.

[4] Arbyn M, de Sanjosé S, Saraiya M, Sideri M, Palefsky J, Lacey C (2012). EUROGIN 2011 roadmap on prevention and treatment of HPV-related disease. Int J Cancer.

[5] Arbyn M, Ronco G, Anttila A, Meijer CJ, Poljak M, Ogilvie G (2012). Evidence regarding human papillomavirus testing in secondary prevention of cervical cancer. Vaccine.

[6] Gök M, Heideman DA, van Kemenade FJ, Berkhof J, Rozendaal L, Spruyt JW (2010). HPV testing on self collected cervicovaginal lavage specimens as screening method for women who do not attend cervical screening: cohort study. BMJ.

[7] Marquardt K, Büttner HH, Broschewitz U, Barten M, Schneider V (2011). Persistent carcinoma in cervical cancer screening: non-participation is the most significant cause. Acta Cytol.

[8] Enerly E, Bonde J, Schee K, Pedersen H, Lönnberg S, Nygård M (2016). Self-sampling for human papillomavirus testing among non-attenders increases attendance to the norwegian cervical Cancer Screening Programme. PLoS ONE.

[9] Nelson EJ, Maynard BR, Loux T, Fatla J, Gordon R, Arnold LD (2017). The acceptability of self-sampled screening for HPV DNA: a systematic review and meta-analysis. Sex Transm Infect.

[10] Deeny LQM, Sankaranarayanan R (2006). Chapter 8: screening for cervical cancer in developing countries. Vaccine.

[11] Poljak M, Oštrbenk Valenčak A, Gimpelj Domjanič G, Xu L, Arbyn M. Commercially available molecular tests for human papillomaviruses: a global overview. Clinical microbiology and infection: the official publication of the European Society of Clinical Microbiology and Infectious Diseases. 2020. 10.1016/j.cmi.2020.03.033 32247892

[12] Hawkes D, Keung MHT, Huang Y, McDermott TL, Romano J, Saville M (2020). Self-Collection for Cervical Screening Programs: from research to reality. Cancers.

[13] Leinonen MK, Schee K, Jonassen CM, Lie AK, Nystrand CF, Rangberg A (2018). Safety and acceptability of human papillomavirus testing of self-collected specimens: a methodologic study of the impact of collection devices and HPV assays on sensitivity for cervical cancer and high-grade lesions. J Clin Virol.

[14] Belinson JL, Du H, Yang B, Wu R, Belinson SE, Qu X (2012). Improved sensitivity of vaginal self-collection and high-risk human papillomavirus testing. Int J Cancer.

[15] Arbyn M, Smith SB, Temin S, Sultana F, Castle P (2018). Detecting cervical precancer and reaching underscreened women by using HPV testing on self samples: updated meta-analyses. BMJ.

[16] Arbyn M, Peeters E, Benoy I, Vanden Broeck D, Bogers J, De Sutter P (2018). VALHUDES: a protocol for validation of human papillomavirus assays and collection devices for HPV testing on self-samples and urine samples. J Clin Virol.

[17] Bennett KM, William B, Coleman GJT (2017). Molecular Testing for Human Papillomaviruses.

[18] Brukner I, Eintracht S, Papadakis AI, Faucher D, Lamontagne B, Spatz A (2020). Maximizing confidence in a negative result: quantitative sample adequacy control. J Infect Public Health.

[19] Jentschke M, Chen K, Arbyn M, Hertel B, Noskowicz M, Soergel P (2016). Direct comparison of two vaginal self-sampling devices for the detection of human papillomavirus infections. J Clin Virol.

[20] Chen K, Ouyang Y, Hillemanns P, Jentschke M (2016). Excellent analytical and clinical performance of a dry self-sampling device for human papillomavirus detection in an urban chinese referral population. J Obstet Gynaecol Res.

[21] Snijders PJ, Verhoef VM, Arbyn M, Ogilvie G, Minozzi S, Banzi R (2013). High-risk HPV testing on self-sampled versus clinician-collected specimens: a review on the clinical accuracy and impact on population attendance in cervical cancer screening. Int J Cancer.

[22] Jentschke M, Soergel P, Hillemanns P (2013). Evaluation of a multiplex real time PCR assay for the detection of human papillomavirus infections on self-collected cervicovaginal lavage samples. J Virol Methods.

[23] Arbyn M, Verdoodt F, Snijders PJ, Verhoef VM, Suonio E, Dillner L (2014). Accuracy of human papillomavirus testing on self-collected versus clinician-collected samples: a meta-analysis. Lancet Oncol.

[24] Poljak M, Kocjan BJ, Oštrbenk A, Seme K (2016). Commercially available molecular tests for human papillomaviruses (HPV): 2015 update. J Clin Virol.

[25] Bell M, Verberckmoes B, Devolder J, Vermandere H, Degomme O, Guimarães YM et al. Comparison between the Roche Cobas 4800 human papillomavirus (HPV), Abbott RealTime High-Risk HPV, Seegene Anyplex II HPV28, and Novel Seegene Allplex II HPV28 assays for high-risk HPV detection and genotyping in mocked self-samples. Microbiol Spectr.0:e00081–23. 10.1128/spectrum.00081-23 PMC10433804 37284753

