# Supplementary material for: Evaluation of the applicability of internal controls on self-collected samples for high-risk human papillomavirus is needed
Source: BMC Womens Health. 2023 Nov 27;23:635. doi: 10.1186/s12905-023-02691-8 (PMC10683318; doi:10.1186/s12905-023-02691-8)
Supplement: Supplementary file 1 — Supplementary Material 1 [file 12905_2023_2691_MOESM1_ESM.docx]

**Supplementary table 1.** Samples reported as ‘negative’ by Abbott assay with a detectable signal for hr-HPV with CN above the cut-off for positivity^a^ (= not detected, but amplified (NDBA^b^))

| UZ Ghent | | | | | AML |
| --- | --- | --- | --- | --- | --- |
|  | Result reported | HPV 16 | HPV 18 | HPV other |  |
| 1 | NDBA^b^ |  |  | 34,52 |  |
| 2 | NDBA |  |  | 32,24 |  |
| 3 | NDBA |  |  | 32,08 |  |
| 4 | NDBA |  |  | 33,02 |  |
| 5 | NDBA |  |  | 35,19 |  |
| 6 | NDBA |  |  | 35,4 |  |
| 7 | NDBA | 35,31 |  |  |  |
| 8 | NDBA |  |  | 34,62 | HPV 67^c^ |
| 9 | NDBA | 33,68 |  |  |  |
| 10 | NDBA |  |  | 32,22 | HPV 52 |
| 11 | NDBA |  |  | 34,73 |  |
| 12 | NDBA |  |  | 35,88 |  |
| 13 | NDBA |  |  | 34,9 | HPV 67 ^c^ |
| 14 | NDBA |  |  | 33,45 |  |
| 15 | NDBA |  | 34,59 |  |  |
| 16 | NDBA |  |  | 35,44 |  |

^a^The cut-off value is <32.00

^b^NDBA (not detected but amplified): phenomenon for the Abbott RealTime assay in which the assay reports samples to be negative despite showing an amplification curve with a CN value higher than the positivity cut-off

^c^Low risk HPV type not included in the Abbott RealTime assay
